# A functionalist approach to online trolling

**DOI:** 10.3389/fpsyg.2023.1211023

**Published:** 2023-10-10

**Authors:** Lewis Nitschinsk, Stephanie J. Tobin, Eric J. Vanman

**Affiliations:** ^1^School of Psychology, The University of Queensland, Brisbane, QLD, Australia; ^2^School of Psychology and Counselling, Queensland University of Technology, Brisbane, QLD, Australia

**Keywords:** trolling, sadism, psychopathy, anonymity, online, motivations

## Abstract

Online trolling is often linked to sadism and psychopathy. Yet, little research has assessed why people high in these traits seek online environments to achieve their nefarious goals. We employ a functionalist approach to examine whether people high in sadism and psychopathy are motivated to seek the affordances of online environments (e.g., anonymity) to reveal their malevolent self-aspects by engaging in trolling behavior. A sample of 515 university undergraduates (*M*_age_ = 20.47) read vignettes depicting trolling incidents and rated the acceptability of the perpetrators’ actions and whether they had ever written similar comments. Participants then completed measures of psychopathy, sadism, and toxic anonymous motivations. We find that toxic anonymous motivations partially mediate the relationship between psychopathy and sadism, and online trolling. Whereas trolling is often understood through its underlying personality traits, toxic motivations to seek anonymity may be a more proximal predictor of who is likely to troll online.

## Introduction

Online trolling is a behavior that deliberately attempts to deceive, aggress, or disrupt others on the Internet (Buckels et al., 2019; Cook et al., 2019). The behavior often intends to trigger or antagonize other users for their own entertainment (Nitschinsk et al., 2022b). People who troll online are typically anonymous and do not know the person they are targeting. Trolling is most commonly associated with sadism and psychopathy (Buckels et al., 2014, 2019; Craker and March, 2016). It is also associated with situational factors, including mood, discussion context, and feelings of anonymity (Cheng et al., 2017; Nitschinsk et al., 2022b).

Anonymous situations can often lead people to behave differently than when they are identifiable (Zimbardo, 1969; Lea et al., 1992; Douglas and McGarty, 2001). As a result, anonymity can be advantageous, so some people seek online environments—which can afford users anonymity—to achieve their goals (Nitschinsk et al., 2021). Functionalist theories of personality and social behavior suggest that certain features of a person (e.g., individual differences) serve as inputs for agendas (e.g., motivations), which then lead to specific outcomes (Snyder and Cantor, 1998). The potentially gratifying outcomes of anonymity could benefit those with psychopathic or sadistic tendencies. People high in these traits may be motivated to seek anonymity online to pursue their toxic goals. Here, we use this functionalist approach to assess whether toxic motivations to seek anonymity mediate the relationship between psychopathy/sadism and self-reported trolling acceptance/perpetration.

Traits such as psychopathy (impulsivity and a callous lack of empathy) and sadism (a desire to harm other people for pleasure) are most associated with trolling (Buckels et al., 2019). However, trolling is also associated with other factors, including social dominance orientation, a lack of affective empathy, and dysfunctional impulsivity (Buckels et al., 2014; March et al., 2017; Sest and March, 2017; Bentley and Cowan, 2021). People troll for multiple reasons. These include revenge, thrill-seeking, and boredom (Shachaf and Hara, 2010; Cook et al., 2018; Pfattheicher et al., 2021). Trolling can also be viewed as humorous to the perpetrator and observers in online environments, which may further perpetuate the behavior (Cook et al., 2018; Sanfilippo et al., 2018).

Some researchers have suggested that anonymity facilitates trolling (Griffiths, 2014; Coles and West, 2016; Cook et al., 2018). However, research has not investigated how the perceived benefits of anonymity influence trolling on the Internet. Anonymity—a continuum ranging from unidentifiability to identifiability—is an affordance of online environments (Christopherson, 2007; Evans et al., 2017). Certain aspects of online environments (e.g., asynchronicity and reduced visual cues) make people feel more anonymous (Suler, 2004; Lapidot-Lefler and Barak, 2012). This can lead to feelings of disinhibition, whereby people feel less constrained by social norms (Christie and Dill, 2016; Stuart and Scott, 2021). Feeling anonymous can influence online behavior, leading to self-beneficial (McKenna and Bargh, 2000; Joinson, 2001) and negative social outcomes (Zimmerman and Ybarra, 2016). For example, the disinhibiting effects of anonymity increased trolling in an experimental online chat room (Nitschinsk et al., 2022b).

According to the Uses and Gratifications approach, people seek environments where they can pursue their goals (Rubin, 2009). Some people may be motivated to seek online environments that afford users anonymity. For instance, anonymous online environments may make it easier to aggravate others and manipulate people while remaining unaccountable for their actions (Griffiths, 2014; Nitschinsk et al., 2022a). As a result, some people may be motivated to seek online anonymity to reveal certain malevolent self-aspects and engage in toxic behaviors like trolling. Further, because trolling is sometimes celebrated or viewed as humorous by Internet communities, this may motivate people to seek out online environments to troll (Sanfilippo et al., 2018).

### The present research

Using a functionalist approach, we tested whether psychopathy and sadism are associated with toxic anonymous motivations that increase trolling perpetration and beliefs that trolling is acceptable and appropriate. To assess trolling acceptance and perpetration, participants read a series of vignettes where one person trolled another online. Participants rated how acceptable and appropriate the perpetrator’s actions were and whether they had ever written a comment similar to the perpetrator in each scenario. To assess individual differences, we had participants complete measures of psychopathy, sadism, Machiavellianism, narcissism, global trolling, and motivations to seek anonymity.

Assuming the above causal model, we predicted that toxic anonymous motivations would explain the previously observed associations between psychopathy, sadism, and trolling. Of course, statistical tests are conditional on the tested model’s validity and cannot identify a true causal model without recognizing the possibility of alternative explanations (Fiedler et al., 2018). Therefore, we included alternative dark traits (i.e., Machiavellianism, narcissism) as covariates to rule out some potential alternative explanations. Additionally, we tested whether alternative motivations to seek anonymity mediated the above relationships.

## Methods

An institutional ethics board approved this study. We report how we determined our sample size, all data exclusions, all manipulations, and all measures in the study. All data, analysis code, and research materials are available on OSF and can be accessed at: https://osf.io/jc386/?view_only=2b9038ab5e4d470297ff208035ac028e. The predicted associations were preregistered,^1^ but it was decided later that the mediational model was the best way to test the associations.

### Participants

We recruited participants via the student pool of an Australian university in exchange for course credit. The initial sample included 565 participants. We excluded 50 people due to incomplete responses. Therefore, the final sample included 515 participants (women = 332, men = 177, non-binary = 1, prefer not to say = 5; age range = 17–53, *M*_age_ = 20.47, *SD* = 4.33). Of these, 79% of participants’ primary language was English, 16% was Chinese, and 5% other.

### Measures

We used the 16-item Online Anonymity Questionnaire (OAQ) to assess motivations to behave toxically or reveal malevolent self-aspects in anonymous online environments (e.g., “*I like being anonymous because I can say whatever I want without consequences”;* Nitschinsk et al., 2021). The OAQ also assesses motivations to self-express while anonymous, all items for the OAQ are included in the supplemental materials. We used the 12-item iTroll questionnaire to assess global trolling (e.g., “*I consider myself to be a troll”;* Buckels et al., 2019). To assess trait psychopathy, along with narcissism and Machiavellianism, we used the 27-item Short Dark Triad (SD3; Jones and Paulhus, 2014). We assessed the OAQ, Itroll, and SD3 on a 5-point scale ranging from 1 (*Strongly Disagree*) to 5 (*Strongly Agree*). Finally, to assess trait sadism, we used the 10-item Short Sadistic Impulse Scale (SSIS), measured on a 2-point scale (1 = *disagree*, 2 = *agree*, O’Meara et al., 2011, for scale reliabilities and descriptive statistics, see Table 1). Correlations between anonymous self-expression, narcissism, Machiavellianism, trolling attitudes, and trolling perpetration are included in Supplementary Table A of the supplemental materials. We did not include results from these variables in this paper as they are not focal predictors.

**Table 1:** Descriptive statistics and bivariate correlations.

	*M (SD)*	1	2	3	4	5	6
Trolling acceptance	2.53 (0.62)	*0.81*					
Trolling perpetration	2.03 (0.81)	0.59***	*0.69*				
Toxic anonymity	1.98 (0.89)	0.37***	0.51***	*0.85*			
Psychopathy	2.02 (0.60)	0.41***	0.50***	0.57***	*0.74*		
Sadism	1.10 (0.14)	0.28***	0.30***	0.37***	0.36***	*0.66*	
Global trolling	2.04 (0.66)	0.40***	0.39***	0.47***	0.41***	0.25***	*0.87*

*N* = 515, *N* = 494 for correlations involving the trolling perpetration. Cronbach’s alphas are included in italics on the diagonal. *p < 0.05, **p < 0.01, ***p < 0.001.

### Procedure

After providing consent, participants completed demographic questions regarding their age, gender, and language. Next, participants read scenarios that depicted an interaction between two people in an online environment. Each scenario described an online interaction between two strangers where one person (the perpetrator) responded to another person’s comment or actions. The scenarios occurred in various online contexts, including social media or playing an online game. The perpetrator responded by trolling the individual or responding with a benign message. The research team formulated each trolling scenario, following the definition that trolling is a deliberate attempt to deceive, aggress, or disrupt others on the Internet (Buckels et al., 2019; Cook et al., 2019). Twenty-seven scenarios were created, with participants randomly viewing one of three scenario sets. The scenario sets accounted for different types of deceptive, aggressive, and disruptive forms of trolling. Each set contained nine scenarios, with three scenarios depicting trolling on social media, three scenarios depicting trolling in an online game, and three scenarios depicting a benign interaction not to raise suspicion of the study’s aims. All perpetrators in the scenario were given male, Western-centric names to hold constant other demographic factors that could influence responses. The complete list of scenarios is included in the supplemental materials.

An example of the wording of a scenario that is deceptive in nature is:

*Dylan was playing a multiplayer online game with strangers on Friday night. Over the chat Dylan told a member of his team to head right, however, the team member instead decided to go left and was promptly killed, meaning they lost the match. At the conclusion of the game Dylan wrote, “Wow, you are actually my hero, great decision bud, keep up the good work.”*

For each scenario, participants rated how acceptable and appropriate they viewed the perpetrator’s comments on a 5-point scale (1 = *Very Unacceptable/Inappropriate,* 5 = *Very Acceptable/Appropriate*), and if they had ever written a comment like the perpetrator’s while online, again using a 5-point scale (1 = *Definitely Not,* 5 = *Definitely Yes*). Participants could skip the question or answer “not applicable” if they did not play multiplayer online games or use social media. Finally, participants completed a questionnaire package that included the OAQ, SD3, Itroll, and SSIS.

### Data analysis

We analyzed all data using R version 4.2.1 (R Core Team, 2022). We performed four mediational analyses to test our hypothesis that toxic motivations for seeking anonymity mediate the relationship between sadism/psychopathy and trolling acceptance/perpetration. A bootstrapping method of the standard errors was used with 5,000 repeated samples. Each model also included Machiavellianism, narcissism, and either psychopathy or sadism as covariates. We also ran additional mediational analyses that are reported in the supplemental materials. First, we ran the above four mediational analyses without covariates. We also ran the above mediational analyses controlling for the scenario set that the participants viewed. Finally, to assess an alternative mediator, we performed the above mediational analyses to determine whether self-expression motives for seeking anonymity mediate the relationship between sadism/psychopathy and trolling acceptance/perpetration.

## Results

### Variable creation

We created the variable “trolling acceptance” by averaging scores of how acceptable and appropriate participants viewed the six trolling vignettes. Trolling acceptability and appropriateness were highly correlated (*r* = 0.84, *p* < 0.001), indicating that the variables can be combined. We created the variable “trolling perpetration” by averaging the scores of whether participants had written a similar comment to the perpetrator in the six trolling vignettes. For trolling perpetration, 16.6% of responses were answered as not applicable. To accommodate these missing data, trolling perpetration was only calculated for participants who responded to at least half of the items on trolling perpetration.

### Correlation

Trolling acceptance was positively associated with trolling perpetration. Further, trolling acceptance and perpetration were positively related to toxic anonymity, psychopathy, sadism, and global trolling (see Table 1).

### Mediational analysis

**Figure 1:**
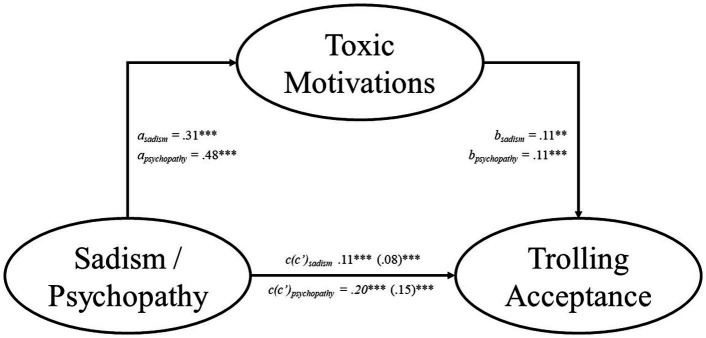
Toxic anonymous motivations partially mediate the relationship between sadism/psychopathy and trolling acceptance. We report the standardized coefficients for all variables. Each model included Machiavellianism, narcissism, and either psychopathy or sadism as covariates. The numbers in the brackets indicate the direct effect of each model. **p < 0.01, ***p < 0.001.

**Figure 2:**
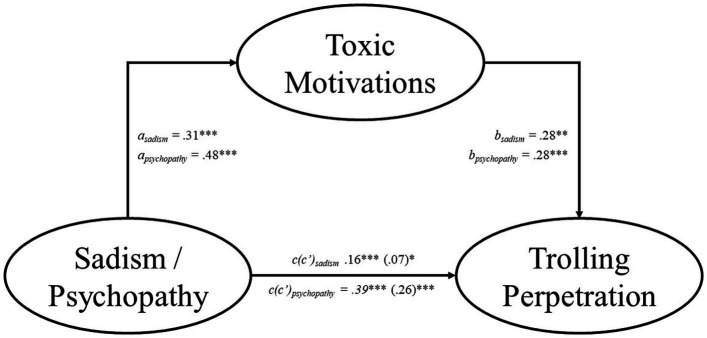
Toxic anonymous motivations partially mediate the relationship between sadism/psychopathy and trolling perpetration. We report the standardized coefficients for all variables. Each model included Machiavellianism, narcissism, and either psychopathy or sadism as covariates. The numbers in the brackets indicate the direct effect of each model. *p < 0.05, **p < 0.01, ***p < 0.001.

The results indicate that the effects of sadism and psychopathy on trolling acceptance and perpetration were partially mediated by toxic motivations (see Figures 1, 2). Specifically, the total effect of sadism was significant for both trolling acceptance (total effect = 0.11, *SE* = 0.03, *p* < 0.001, 95% CI [0.07, 0.16]) and trolling perpetration (total effect = 0.16, *SE* = 0.03, *p* < 0.001, 95% CI [0.09, 0.24]). The indirect effect of sadism via toxic motivations was significant for both trolling acceptance (indirect effect = 0.03, *SE* = 0.03, *p* < 0.001, 95% CI [0.02, 0.06]) and trolling perpetration (indirect effect = 0.09, *SE* = 0.04, *p* < 0.001, 95% CI [0.06, 0.12]). The direct effect was significant between sadism and both trolling acceptance (direct effect = 0.08, *SE* = 0.03, *p* < 0.001, 95% CI [0.03, 0.12]) and trolling perpetration (direct effect = 0.07, *SE* = 0.03, *p* = 0.028, 95% CI [0.01, 0.14]).

The total effect of psychopathy was significant for both trolling acceptance (total effect = 0.20, *SE* = 0.03, *p* < 0.001, 95% CI [0.14, 0.26]) and trolling perpetration (total effect = 0.39, *SE* = 0.04, *p* < 0.001, 95% CI [0.30, 0.48]). The indirect effect of psychopathy via toxic motivations was significant for both trolling acceptance (indirect effect = 0.05, *SE* = 0.03, *p* < 0.001, 95% CI [0.03, 0.08]) and trolling perpetration (indirect effect = 0.13, *SE* = 0.04, *p* < 0.001, 95% CI [0.09, 0.18]). The direct effect was significant between psychopathy and both trolling acceptance (direct effect = 0.15, *SE* = 0.03, *p* < 0.001, 95% CI [0.09, 0.21]) and trolling perpetration (direct effect = 0.26, *SE* = 0.04, *p* < 0.001, 95% CI [0.17, 0.33]).

We achieved the same direct and indirect effects for mediational analyses without covariates and when controlling for the scenario. Finally, we assessed the alternate pathway of anonymous self-expression motivations mediating the relationship between psychopathy and sadism and trolling acceptance and perpetration. For each model, the indirect effect was not significant, indicating no mediation (see Supplementary Tables A–M for full results).

## Discussion

Using a functionalist approach, we show that people with sadistic and psychopathic tendencies are motivated to seek anonymity, allowing them to troll other people more easily in online environments. Past research has found both sadism and psychopathy to be strong predictors of trolling (Buckels et al., 2014; Seigfried-Spellar and Lankford, 2018; March and Steele, 2020), and some researchers have speculated that the perceived gratifications of anonymous environments facilitate trolling (Griffiths, 2014; Coles and West, 2016; Cook et al., 2018; Sanfilippo et al., 2018). However, we extended these findings by empirically showing a potential mechanistic link between individual differences and trolling behavior.

Importantly, we offer further theoretical insight into why people high in sadism and psychopathy engage in online trolling. Specifically, we found that people more likely to troll or view trolling as acceptable and appropriate may be motivated to seek out anonymous environments to behave toxically. Sadism and psychopathy are typified by a desire to behave cruelly or maliciously toward others. However, revealing these malevolent self-aspects would likely lead to negative social ramifications. Anonymity allows people to behave however they want, with little fear of reprisal, so trolls seek these environments to pursue their goals (Cook et al., 2019; Bentley and Cowan, 2021).

These results are conditional, as a statistical test cannot establish a true causal model. To rule out theoretically plausible models, we controlled for Machiavellianism and narcissism. We also assessed alternative mediators, including motivations to seek anonymity online to self-express. These models were not significant. It is possible, however, that other unmeasured variables have generated these empirical patterns. Since this research did not use an experimental design, we cannot rule out these alternate hypotheses. Nonetheless, our basis for assessing these mediational effects stems from a solid theoretical basis. Therefore, the findings of this study are consistent with the idea that toxic anonymous motivations can provide a more proximal predictor of who is likely to endorse and perpetrate trolling.

### Implications

Our research contributes to the trolling literature by offering a plausible mechanistic link between individual differences and trolling. Some have criticized the trolling literature for suggesting that the behavior has a single personality-driven cause (Cook et al., 2018). Individual differences are associated with trolling; however, we exemplify how anonymity—an affordance of online environments—may benefit people who troll. As such, they are motivated to seek out anonymous environments. Understanding why people with malevolent personality traits are motivated to seek online environments is useful in developing future interventions to reduce trolling and improve online civility. Additionally, we used hypothetical vignettes to assess trolling acceptance and perpetration, an approach seldom used in the trolling literature (Bentley and Cowan, 2021). An advantage of vignette designs is that they provide more realistic scenarios, thus increasing the generalizability of the results (Aguinis and Bradley, 2014). Further, the positive associations between responses to these vignettes and self-report measures of global trolling indicate the viability of such designs.

### Limitations and future directions

Our study only included male western-centric names for perpetrators of trolling in our hypothetical vignettes. It is possible that the acceptability of trolling may be different across both gender and culture. Second, although our study outlines the importance of anonymity in online environments for trolls, we do not explicitly compare differences in trolling across anonymous and identifiable contexts. This opens exciting opportunities for future research to experimentally compare these differences. Third, our study focused on anonymity as an affordance of online environments. Other affordances—audience size—may also appeal to trolls. As such, future research should assess whether the perceived benefits of these affordances are associated with trolling.

## Conclusion

Trolling is associated with sadism and psychopathy, as these traits often entail the ability to behave cruelly toward others. Trolling is also an online behavior, meaning that online environments’ affordances may also explain why people troll. People seek environments where they can pursue their goals. Online environments may appeal to trolls as they often afford anonymity, allowing them to behave malevolently with little fear of reprisal. Our research indicates that motivations to seek anonymity to behave toxically are a more proximal predictor of who is likely to troll.

## Data availability statement

The datasets presented in this study can be found in online repositories. The names of the repository/repositories and accession number(s) can be found in the article/Supplementary material.

## Ethics statement

The studies involving human participants were reviewed and approved by the Health and Behavioral Sciences Low and Negligible Research Committee. The patients/participants provided their written informed consent to participate in this study.

## Author contributions

LN: conceptualization, methodology, formal analysis, investigation, writing—original draft, writing—reviewing and editing, visualization. ST: formal analysis, writing—review and editing, supervision. EV: conceptualization, methodology, writing—reviewing and editing, supervision. All authors contributed to the article and approved the submitted version.

## Footnotes

^1^ https://osf.io/3yqev/?view_only=b18c178208f94d98a0167fa9550461da
