# Supplementary material for: A functionalist approach to online trolling
Source: Front Psychol. 2023 Oct 10;14:1211023. doi: 10.3389/fpsyg.2023.1211023 (PMC10598604; doi:10.3389/fpsyg.2023.1211023)
Supplement: Supplementary file 1 [file Table_1.DOCX]

**Supplemental Materials**

**The Online Anonymity Questionnaire**

This questionnaire is focused on why people are sometimes anonymous on the Internet. Please respond to the statements on anonymity below by indicating how much you agree/disagree with each.

1. I feel more comfortable disclosing information about my ideas, thoughts, and feelings when I am anonymous.
2. Being anonymous allows me to share thoughts and feelings I otherwise wouldn’t share with people who know me.
3. Being anonymous allows me to experiment with new ideas.
4. I feel like I can be somebody else when I am anonymous.
5. I can present myself in a different way when I’m anonymous.
6. Being anonymous online allows me to escape or distract myself from reality.
7. When I am anonymous online, I can talk to people who wouldn’t normally talk to me in the offline world.
8. Being anonymous allows me to join groups I wouldn’t normally join in the real world.
9. I can connect with people I normally wouldn’t when I’m anonymous.
10. I feel like I can express my true self when I am anonymous.
11. I am more likely to do things that are unlawful or illegal when I am anonymous.
12. When I’m anonymous I do things that are normally unacceptable in society.
13. I get satisfaction from aggravating people anonymously online.
14. Being anonymous online is fun because I don’t get in trouble for what I say.
15. When I am anonymous, I find it easier to trick and manipulate people.
16. I like being anonymous because I can say whatever I want without consequences.

**Scoring Procedure**

Anonymous Self-Expression: 10 Items

1, 2, 3, 4, 5, 6, 7, 8, 9, 10

Anonymous Toxicity: 6 Items

11, 12, 13, 14, 15, 16

Scale Ranges from 1 (strongly disagree) to 5 (strongly agree)

No reverse scoring on any items

**Section One – Scenario Sets**

***Benign Interactions***

1. Tyler was playing a multiplayer online game with strangers on the weekend. He could see that one of the players on his team was new and had no idea what he was doing. After losing the first two rounds, Tyler wrote in the chat “Hey man, this round just follow me the whole time, it’s not too hard you’ll get the hang of it”. The player then followed Tyler’s character the entire third game.
2. Charlie was browsing Facebook on Friday night when he came across a post by someone showing off photos from a recent hiking trip. Charlie then commented “These photos are awesome! Where were they taken?”.
3. Arlo was browsing Facebook on a Friday night when he came across a post by someone detailing their recent holiday to Fiji. Arlo then commented on the post “Hey these photos are awesome! Seems like a fun holiday”.

***Set A – Trolling Scenarios***

1. Alex was playing an online game with strangers on Friday night. One member of his team seemed new and was unsure how to play the game properly, they spent most of the game in the corner of the map not contributing. Alex then wrote in the chat “Hey, if you don’t know how to play the game then get the f#ck off my team”. The player promptly logged off.
2. Joel was browsing Facebook on a Friday night when he came across a post by someone detailing their recent holiday to Fiji. Joel then commented on the post “Hey these photos are f*^#ing terrible, why even post them”. The post was promptly deleted.
3. Dylan was playing an online game with strangers on Friday night. Over the chat Dylan told a member of his team to head right, however the team member instead decided to go left and was promptly killed, meaning they lost the match. At the conclusion of the game Brad wrote “Wow, you’re actually my hero, great decision their bud, keep up the good work”.
4. Aiden was browsing Facebook on a Friday night when he came across a misinformed comment underneath a news article. Aiden replied to the comment saying “Hey, wow, great comment! Glad to see we’ve got an expert in the mix!” The comment was promptly deleted.
5. Brayden was playing an online game with strangers on Friday night. In the final moments of the game a member of his team made a crucial mistake, leading to his team losing. At the conclusion of the game Brayden wrote in the chat “H-hey buddy, do you remember me from Biology? Freshman year? It’s Laura. I just wanted to stop by since you missed the last reunion. I was look for you. I always thought you were really smart and talented, but I could never work up the nerve to tell you. Anyway, I hope you’re doing well…HAHA just kidding, it’s still Brayden and you still suck at playing this game. Anyway, the gym awaits, see ya man good talk”. The player promptly logged off.
6. Nathan was browsing Facebook on a Friday night when he came across a post detailing someone’s new gym routine. Nathan then started spamming “L0L” in the comment section, so that no one else’s comments could be seen. The post was promptly deleted.

***Set B – Trolling Scenarios***

1. Brayden was playing an online game with strangers on Friday night. In the final moments of the game a member of his team made a crucial mistake, leading to his team losing. At the conclusion of the game Brayden wrote in the chat “Hey if you’re going to make stupid mistakes like that then get the f#ck off my team”. The player promptly logged off.
2. Nathan was browsing Facebook on a Friday night when he came across a post detailing someone’s new gym routine. Nathan then commented on the post “Hey what the f#ck is this? Do you even know how to work out? This sucks”. The post was promptly deleted.
3. Alex was playing an online game with strangers on Friday night. One member of his team seemed new and was unsure how to play the game properly, they spent most of the game in the corner of the map not contributing. At the end of the game Alex wrote in the chat “Hey, wow, great game bud! Glad to see we’ve got an expert in the mix!” The player promptly logged off.
4. Joel was browsing Facebook on a Friday night when he came across a post by someone detailing their recent holiday to Fiji. Joel then commented on the post “Good job bud, real MVP with the photos, I can see you’re a bit of a sharpshooter.” The post was promptly deleted.
5. Dylan was playing an online game with strangers on Friday night. Over the chat Dylan told a member of his team to head right, however the team member instead decided to go left and was promptly killed, meaning they lost the match. At the conclusion of the game Brad started spamming “L0L” in the chat, meaning no one could message each other and discuss tactics for the next game. That player promptly logged off.
6. Aiden was browsing Facebook on a Friday night when he came across a misinformed comment underneath a news article. Aiden replied to the comment saying “My mother said to me- ‘Don’t ever smoke. Please don’t put your family through what your Grandfather put us through.’ I agreed, I made a pact with myself that I would never smoke those vile things. Don’t get me wrong, I had plenty of opportunities at house parties throughout my teen years, but I’m proud to say at 28 I’m married, healthy, happy and I have never touched a cigarette. I must say, I feel a very slight sense for regret for never having done it, because your comment gave me cancer anyway bud”. The comment was promptly deleted.

***Set C – Trolling Scenarios***

1. Dylan was playing an online game with strangers on Friday night. Over the chat Dylan told a member of his team to head right, however the team member instead decided to go left and was promptly killed, meaning they lost the match. At the conclusion of the game Brad wrote “Hey what the f#ck was that? Learn how the play the game or get off our f*^#ing team”. The player promptly logged off
2. Aiden was browsing Facebook on a Friday night when he came across a misinformed comment underneath a news article. Aiden then replied to the comment saying “Hey, if you don’t know what you’re talking about don’t make dumb f*^#ing comments”. The comment was promptly deleted.
3. Brayden was playing an online game with strangers on Friday night. In the final moments of the game a member of his team made a crucial mistake, leading to his team losing. At the conclusion of the game Brayden wrote in the chat “Good job bud, I can see you’re a bit of an MVP, really lead the team to victory.” The player promptly logged off.
4. Nathan was browsing Facebook on a Friday night when he came across a post detailing someone’s new gym routine. Nathan then commented on the post “Wow, you’re actually my hero, great work out their bud, keep up the good work”. The post was promptly deleted.
5. Alex was playing League of Legends on Friday night. One member of his team seemed new and was unsure how to play the game properly, they spent most of the game in the corner of the map not contributing. At the end of the game Alex wrote in the chat “My mother said to me- ‘Don’t ever smoke. Please don’t put your family through what your grandfather put us through.’ I agreed, I made a pact with myself that I would never smoke those vile things. Don’t get me wrong, I had plenty of opportunities at house parties throughout my teen years, but I’m proud to say at 28 I’m married, healthy, happy and I have never touched a cigarette. I must say, I feel a very slight sense for regret for never having done it, because your playstyle gave me cancer anyway bud”. The player promptly logged off.
6. Joel was browsing Facebook on a Friday night when he came across a post by someone detailing their recent holiday to Fiji. Joel then commented on the post “H-hey buddy, do you remember me from Biology? Freshman year? It’s Laura. I just wanted to stop by since you missed the last reunion. I was looking for you. I always thought you were really smart and talented, but I could never work up the nerve to tell you. Anyway, I hope you’re doing well…HAHA just kidding, it’s still Joel and you’re still terrible at taking photos. Anyway, the gym awaits, see ya man good talk”. The post was promptly deleted.

**Section Two – Descriptive Statistics and Correlations**

**Table A**

D*escriptive statistics and bivariate correlations for anonymous self-expression, narcissism, Machiavellianism, trolling acceptance and trolling perpetration.*

|  | *M (SD)* | 1 | 2 | 3 | 4 | 5 |
| --- | --- | --- | --- | --- | --- | --- |
| 1. Trolling Acceptance | 2.53 (0.62) |  |  |  |  |  |
| 1. Trolling Perpetration | 2.03 (0.81) | .59*** |  |  |  |  |
| 1. Anonymous Self-expression | 2.90 (0.85) | .10* | .17*** | *.90* |  |  |
| 1. Narcissism | 2.62 (0.61) | .27*** | .30*** | .16*** | *.72* |  |
| 1. Machiavellianism | 2.81 (0.72) | .23*** | .26*** | .42*** | .41*** | *.81* |

*Note.* *N* = 515, *N = 494* for correlations involving Trolling Perpetration. Cronbach’s alphas are included in italics on the diagonal. Correlations corrected using the Benjamini-Hochberg procedure. **p < .05,* ***p < .01,* ****p < .001.*

**Section Three – Anonymous Toxicity as a Mediator with No Covariates**

**Table B**

*Mediation analysis of trolling acceptance and sadism, mediated by anonymous toxicity.*

|  | Effect | Standard Error | 95% CI |
| --- | --- | --- | --- |
| Total | 0.14*** | 0.04 | [0.11 – 0.20] |
| Indirect | 0.06*** | 0.03 | [0.04 – 0.08] |
| Direct | 0.09*** | 0.03 | [0.05 – 0.14] |

****p < .001*

**Table C**

*Mediation analysis of trolling perpetration and sadism, mediated by anonymous toxicity.*

|  | Effect | Standard Error | 95% CI |
| --- | --- | --- | --- |
| Total | 0.22*** | 0.03 | [0.14 – 0.30] |
| Indirect | 0.12*** | 0.03 | [0.08 – 0.16] |
| Direct | 0.10** | 0.03 | [0.03 – 0.17] |

***p < .01,* ****p < .001*

**Table D**

*Mediation analysis of trolling acceptance and psychopathy, mediated by anonymous toxicity.*

|  | Effect | Standard Error | 95% CI |
| --- | --- | --- | --- |
| Total | 0.22*** | 0.02 | [0.18 – 0.27] |
| Indirect | 0.06*** | 0.03 | [0.03 – 0.09] |
| Direct | 0.16*** | 0.03 | [0.11 – 0.22] |

****p < .001*

**Table E**

*Mediation analysis of trolling perpetration and psychopathy, mediated by anonymous toxicity.*

|  | Effect | Standard Error | 95% CI |
| --- | --- | --- | --- |
| Total | 0.38*** | 0.03 | [0.31 – 0.48] |
| Indirect | 0.13*** | 0.04 | [0.09 – 0.17] |
| Direct | 0.25*** | 0.04 | [0.17 – 0.32] |

****p < .001*

**Section Four – Anonymous Toxicity as a Mediator with Scenario Sets as a Covariate**

**Table F**

*Mediation analysis of trolling acceptance and sadism, mediated by anonymous toxicity.*

|  | Effect | Standard Error | 95% CI |
| --- | --- | --- | --- |
| Total | 0.09*** | 0.02 | [0.04 – 0.13] |
| Indirect | 0.04*** | 0.03 | [0.01 – 0.05] |
| Direct | 0.05** | 0.02 | [0.02 – 0.10] |

***p < .01,* ****p < .001*

**Table G**

*Mediation analysis of trolling perpetration and sadism, mediated by anonymous toxicity.*

|  | Effect | Standard Error | 95% CI |
| --- | --- | --- | --- |
| Total | 0.15*** | 0.03 | [0.08 – 0.22] |
| Indirect | 0.08*** | 0.03 | [0.05 – 0.12] |
| Direct | 0.07* | 0.03 | [0.01 – 0.13] |

**p < .05,* ****p < .001*

**Table H**

*Mediation analysis of trolling acceptance and psychopathy, mediated by anonymous toxicity.*

|  | Effect | Standard Error | 95% CI |
| --- | --- | --- | --- |
| Total | 0.19*** | 0.03 | [0.14 – 0.24] |
| Indirect | 0.05** | 0.03 | [0.02 – 0.07] |
| Direct | 0.14*** | 0.03 | [0.08 – 0.20] |

***p < .01, *****p < .001*

**Table I**

*Mediation analysis of trolling perpetration and psychopathy, mediated by anonymous toxicity.*

|  | Effect | Standard Error | 95% CI |
| --- | --- | --- | --- |
| Total | 0.17*** | 0.04 | [0.11 – 0.23] |
| Indirect | 0.06*** | 0.03 | [0.03 – 0.09] |
| Direct | 0.12*** | 0.04 | [0.05 – 0.17] |

****p < .001*

**Section Five – Anonymous Self-Expression as an Alternate Mediator**

**Table J**

*Mediation analysis of trolling acceptance and sadism, mediated by anonymous self-expression.*

|  | Effect | Standard Error | 95% CI |
| --- | --- | --- | --- |
| Total | 0.09*** | 0.02 | [0.04 – 0.13] |
| Indirect | -0.01 | 0.03 | [-0.01 – 0.01] |
| Direct | 0.09*** | 0.03 | [0.05 – 0.14] |

****p < .001*

**Table K**

*Mediation analysis of trolling perpetration and sadism, mediated by anonymous self-expression.*

|  | Effect | Standard Error | 95% CI |
| --- | --- | --- | --- |
| Total | 0.12*** | 0.03 | [0.06 – 0.19] |
| Indirect | 0.01 | 0.03 | [-0.01 – 0.02] |
| Direct | 0.11*** | 0.03 | [0.05 – 0.19] |

****p < .001*

**Table L**

*Mediation analysis of trolling acceptance and psychopathy, mediated by anonymous self-expression.*

|  | Effect | Standard Error | 95% CI |
| --- | --- | --- | --- |
| Total | 0.18*** | 0.04 | [0.13 – 0.25] |
| Indirect | -0.01 | 0.03 | [-0.01 – 0.01] |
| Direct | 0.19*** | 0.03 | [0.13 – 0.25] |

****p < .001*

**Table M**

*Mediation analysis of trolling perpetration and psychopathy, mediated by anonymous self-expression.*

|  | Effect | Standard Error | 95% CI |
| --- | --- | --- | --- |
| Total | 0.38*** | 0.04 | [0.28 – 0.47] |
| Indirect | 0.01 | 0.03 | [-0.01 – 0.03] |
| Direct | 0.36*** | 0.04 | [0.27 – 0.46] |

****p < .001*
